# Polarity in Liquid Crystals Formed by Self-Assembled Umbrella-Shaped Subphthalocyanine Mesogens

**DOI:** 10.1021/acsami.4c01900

**Published:** 2024-05-06

**Authors:** Ahmad Murad, Elias Baron, Martin Feneberg, Maximilian Baumann, Matthias Lehmann, Alexey Eremin

**Affiliations:** † Institute of Physics, Otto von Guericke University, Magdeburg 39106, Germany; ‡ Institute of Organic Chemistry, University of Würzburg, Würzburg 97074, Germany

**Keywords:** organic semiconductors, ferroelectric liquid crystals, nonlinear optics, photovoltaic, subphthalocyanine

## Abstract

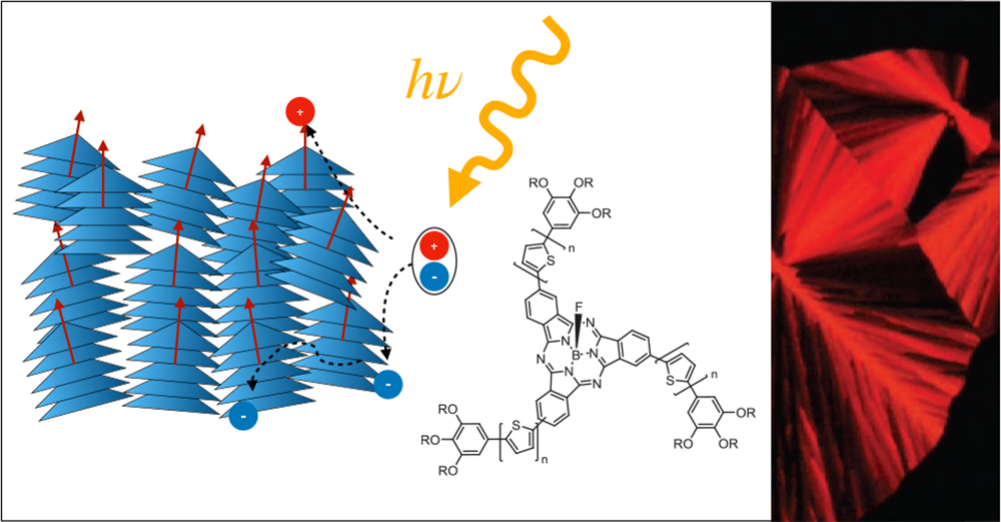

We investigated the properties of p-type semiconducting columnar phases in self-assembled umbrella-shaped mesogens that have subphthalocyanine cores and oligo-thienyl arms. These compounds have nonswitchable phases that exhibit remanent electric polarization and nonlinear optical activity. Additionally, these compounds can generate photocurrents in the visible spectral range due to their wide absorption band. The photocurrent can be significantly increased by doping materials with fullerene. The charge mobility shows an anomalous field dependence, which decreases with the temperature.

## 1 Introduction

With the development of high-resolution television screens, flexible displays, solar cells, and wearable devices, organic electronics are gaining an increasing presence in modern technology and everyday life.^1−3^ However, most semiconducting organic materials/compounds are still strongly competing with inorganic semiconductors regarding charge transport properties.^4,5^ Self-assembly in partially ordered systems such as liquid crystals can provide a framework for improving the electronic properties of such materials, which stimulates intensive research in semiconducting liquid crystals.^6−8^ Polymerisable nematic, smectic, and columnar liquid crystals have been studied for organic light-emitting diodes, field-effect transistors, and photovoltaic applications.^7,8^ Inexpensive spin-coating and printing techniques allow the fabrication of liquid crystal devices efficiently and at low costs.^3^ Another advantage of liquid crystals is the ability to assemble into periodic structures driven by nanosegregation.^8−10^ In ferroelectric liquid crystals also display bulk photovoltaic effect as shown in refs (11,12).

**Figure 1:**
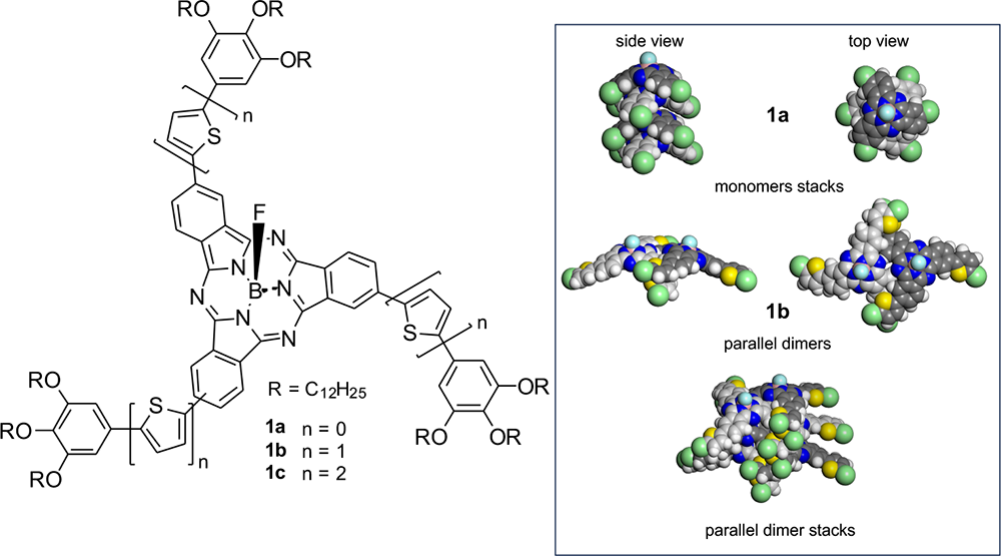
Structure and self-assembly of umbrella-shaped SubPcs **1a**–**1c**. Left: Molecular structure of the SubPcs. Right: Packing in the columnar phases visualized by space-filling representations (gray carbon, white hydrogen, blue nitrogen, yellow sulfur, pink boron, cyan fluorine, and green peripheral aromatic unit). In the columnar phases, mesogens **1b** and **1c** form parallel dimers rotated by 20° about the column axes to avoid steric repulsion. Mesogens of **1a**, however, remain as monomers.

Segregation of incompatible units occurs in amphiphilic molecules composed of chemically different blocks, such as aromatic cores and flexible chains, inducing the formation of periodic structures. In the smectic and columnar phases, the conjugated building blocks segregate in either layers or columns and thus provide 2D or 1D conduction pathways.^7,13^ Large conjugated systems are preferred to maximize the absorption in the visible range of light for the generation of photocurrents. However, large discotic chromophores have high clearing temperatures due to their extended π-conjugated contacts making the required alignment for such applications extremely difficult.^14^ This fact motivates our interest in nonplanar compounds, such as subphthalocyanines (SubPcs). These bowl-shaped chromophores absorb light in the visible range and possess an extremely large dipole moment of up to 14 D.^15^ Depending on the substitution pattern, they can be used as hole or electron carriers and are successfully applied in organic electronics as acceptor or donor materials.^16^ Thus, such materials are promising candidates as substitutes for fullerene acceptors in photovoltaic cells. Only very few thermotropic columnar liquid crystals with SubPc cores are known today.^17−21^ All the derivatives show low clearing temperatures, and owing to the very large dipole moments, they can be aligned in an electric field. Recently the bulk photovoltaic effect could be demonstrated for one of these mesogens, while for others ferroelectric and pyroelectric properties have been reported.^5,18−21^ These intriguing mesomorphic, optical, and electronic properties inspired us to design umbrella-shaped molecules **1a**–**1c** (Figure 1) with a SubPc core, an axial fluorine substituent and conjugated arms of different lengths containing thienyl units.^20^ The small fluorine axial substituent is common in all recent columnar SubPc liquid crystals since it is sufficiently small to promote columnar stacking and increase molecular stability.^22^ The conjugated arms absorb additional UV light and are part of the antenna system, which transfers energy to the core. Further, they are supposed to provide free space to accommodate guest molecules.^23^ We were able to show that all compounds **1a**–**1c** form columnar phases with low clearing temperatures.^23^ Thereby, compound **1a** stacks as a single molecule along the columnar axis, while **1b** and **1c** form dimers with parallel dipoles to reduce the free space between the arms (Figure 1, right). All materials can be aligned in an electric field.

In this paper, we explore in detail polarity and the photovoltaic behavior of the umbrella-shaped mesogens based on SubPc cores and oligo(thienyl) arms **1a**–**1c**. We investigate the character, field, and temperature dependences of the charge carrier mobility and demonstrate the bulk photovoltaic effect. Although the deficiency of charge carriers in the pure compounds results in poor photovoltaic properties, we show that the photovoltaic response can be drastically improved by doping the materials with a small amount of C61 fullerenes as acceptors.

## 2 Experimental Section

### 2.1 Materials

The compounds **1a**–**1c** were synthesized according to a recently published convergent strategy.^20^ The materials were thoroughly purified by recycling gel permeation chromatography. Owing to the synthetic procedure the samples consist of two enantiomeric pairs of molecules, the C_1_ and the C_3_ derivatives, in a ratio of 3:1. These two derivatives are regioisomer, which were not separated to guarantee low transition temperatures. The transition temperatures for the heating and cooling ramps are as follows:

**1a** (heating) Col_h_ 80.2 °C iso; (cooling) iso 77.6 °C Col_h_,
**1b** (heating) glass 74.7 °C Col_h_ 145.8 °C iso; (cooling) iso 142.5 °C Col_h_ 74.7 °C glass,
**1c** (heating) glass 103.0 °C Col_h_ 165.2 °C iso; (cooling) iso 166.8 °C Col_h_ 103.0 °C glass,

where iso designates the isotropic phase and Col_h_ is the hexagonal columnar liquid crystal phase.

### 2.2 Methods

Experimental studies were made in glass cells with transparent sandwich indium tin oxide (ITO) electrodes (E.H.C., Japan). Polarizing microscopy observations were made using an AxioImager D1 microscope (Carl Zeiss GmbH) equipped with the programmable hot stages FS1 (Instec, USA) and LTS200 (Linkam, UK).

Current–voltage characteristics and the photocurrent were measured using a SourceMeter Keithley 2635b (Tektronix/Keithley).

Measurements of optical second harmonic generation (SHG) have been performed using a Nd:YAG laser operating at λ = 1064 nm (10 ns pulse width and 10 Hz repetition rate). The angle of incidence of the primary beam was 30° from the cell normal. The SHG signal was detected in transmission by a photomultiplier tube (Hamamatsu). The acquired signal was calibrated by using a 50 μm reference quartz plate. The SHG interferometry measurements were made by using the same apparatus with an additional quartz reference plate and two counterrotating glass slides. The interferogram was recorded as a function of the tilt angle of the glass plates (see scheme in Figure 6).

Spatially resolved SHG studies were performed using a confocal microscope TCS SP8-Leica. A tunable IR laser (Mai Tai, 780–920 nm) set to λ = 880 nm was used as a fundamental light. As a liquid crystal device, we used 6 μm cells with interdigitated comb electrodes (IPS) and 20 μm electrode separation. An electric field was applied by an arbitrary-wave generator (TTi).

The time-of-flight (ToF) measurements were made with a tunable wavelength laser Q-TUNE-C100-SH from Quantum Light Instruments using an optical parametric oscillator (OPO, 410–2300 nm, 1–100 Hz, pulse energy >1 mJ, pulse duration <1 ns) with second harmonic (SH, 210–410 nm) extension. The OPO system is optically pumped by a diode-pumped solid-state actively Q-switched laser (1064 nm, 20 mJ 100 Hz). The measurements were made on cooling in 5 μm thick ITO cells. All samples were very slowly (0.1 °C/min) cooled from the isotropic temperature to obtain a better homeotropic alignment. In addition, a weak electric field was also applied to the samples, as it helped to achieve a perfect homeotropic orientation of the subphthalocyanine mesogens. The hole mobility was determined from measurements of the transient time τ of the photocurrent as μ_h_ = *d*^2^/(τ*V*), where *V* is the applied voltage and *d* is the cell thickness.

## 3 Results and Discussion

### 3.1 Optical Properties

**Figure 2:**
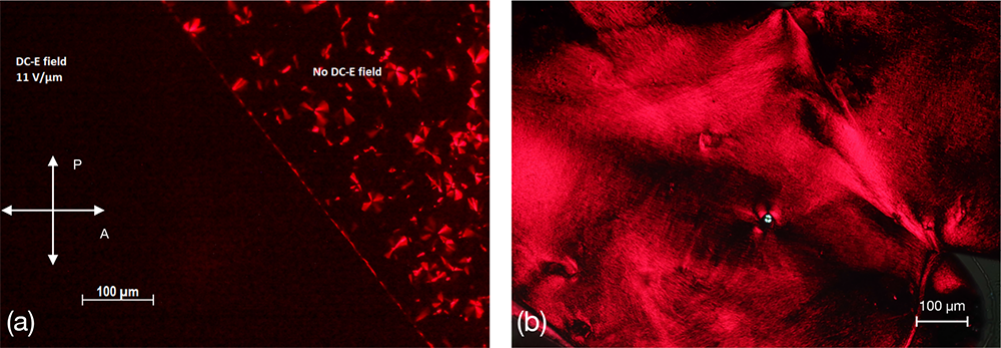
(a) POM image of compound **1b** in a 2.5 μm sandwiched ITO glass cell. The area under the electrodes shows optical extinction, suggesting orthogonal alignment of the optical axis. (b) Optical image of C61-doped compound. The arrows indicate the orientation of the polarizers.

In polarizing optical microscopy (POM), all three compounds in thin cells exhibited randomly oriented birefringent mosaic or pseudofocal conic textures unresponsive to applied electric fields. However, a weak response was observed near the iso-Col_h_ transition. Cooling the samples under a sufficiently high field (≈13 V μm^–1^) resulted in optically extinct polarizing microscopy textures, suggesting a homeotropic (orthogonal) alignment of the molecular stacks (Figure 2). The orthogonal alignment remained even when the external electric field was removed. Inversion of the field polarity did not change the optical appearance, suggesting that the given state was not electrically switchable. Field-induced textural changes could only be observed near the Col_h_-iso transition and were strongly pronounced in cells with in-plane electrodes due to the field-induced alignment.

### 3.2 Polarity and Nonlinear Optics

Generation of the optical second harmonic (SHG) is one of the most remarkable consequences of the polar structure in condensed matter.^24^ This second-order nonlinear effect occurs in noncentrosymmetric media with broken polar symmetry.^25−27^

**Figure 3:**
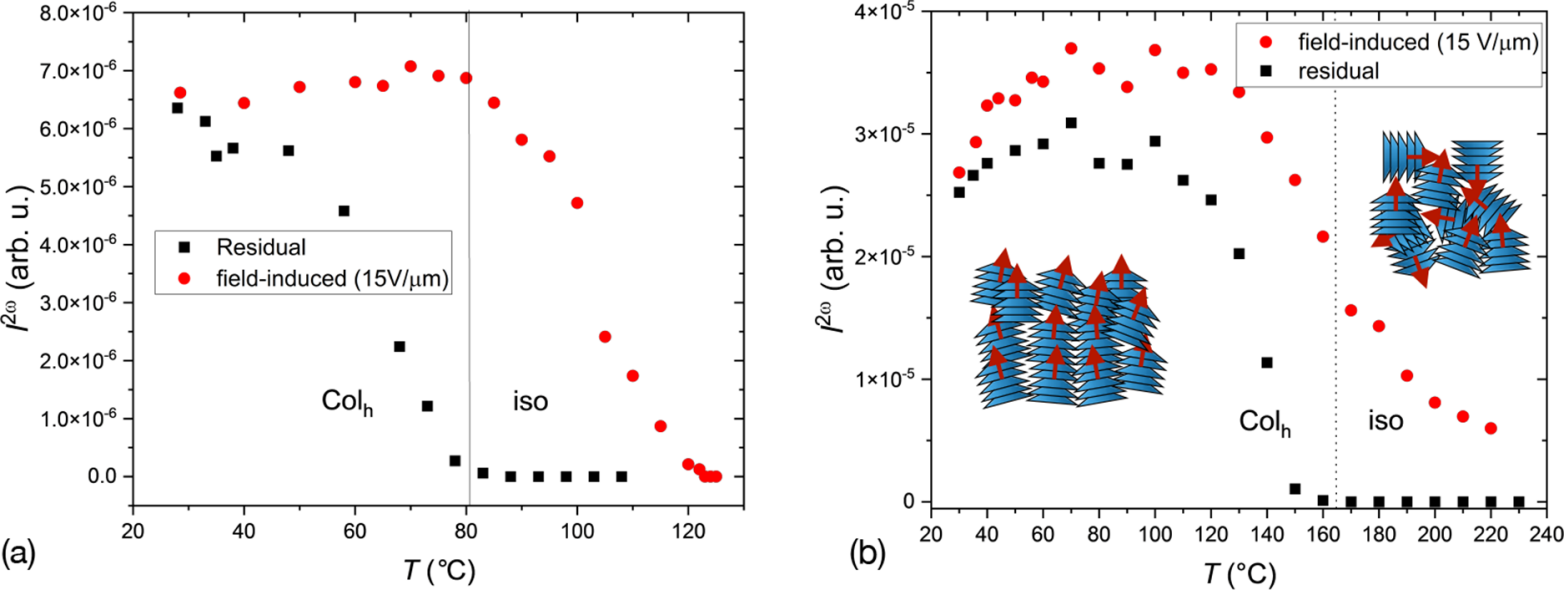
Temperature dependences of the induced (red circles) and residual (black squares) SHG signals for compounds **1a** (a) and **1c** (b). The residual signal was measured while heating samples prepared after cooling in an electric field of 15 V μm^–1^ from the isotropic phase. The electric field was removed after the cooling ramp.

The investigated compounds did not produce any significant SHG signal when no external field was applied. However, when cooled in a DC field from the isotropic phase, SHG activity was observed (Figure 3). The SH signal continued to persist at low temperatures, even after the external field was turned off. The temperature dependencies of the SHG signals for compounds **1a** and **1c** can be seen in Figure 3. In these measurements, the liquid crystals were cooled from the isotropic phase to room temperature under a DC field of 15 V μm^–1^. The DC field was then switched off, and the second measurement of the residual signal was taken while heating.

Remarkably, all of the compounds displayed a field-induced SHG even in the isotropic phase. However, the signal rapidly relaxed at high temperatures when the field was switched off. The field dependence of the induced SHG exhibited a nearly thresholdless behavior near the transition to the isotropic phase and a threshold behavior at lower temperatures. The response to an electric field became much weaker when the materials were cooled far from the Col_h_-iso transition suggesting “freezing” of the polarization dynamics.

**Figure 4:**
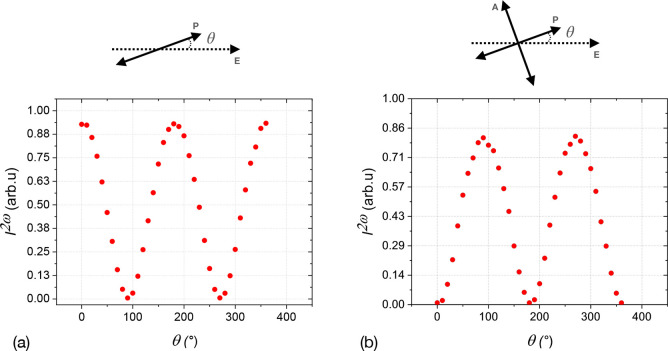
Angular dependence of the SH signal recorded in the field-aligned sample of compound **1b** (9 V μm^–1^) at room temperature (prepared on cooling): (a) between parallel polarizers and (b) between crossed polarizers. The polar axis is aligned horizontally (θ = 0°). P, A, E designate the directions of the polarizer, analyzer, and the electric field, respectively.

**Figure 5:**
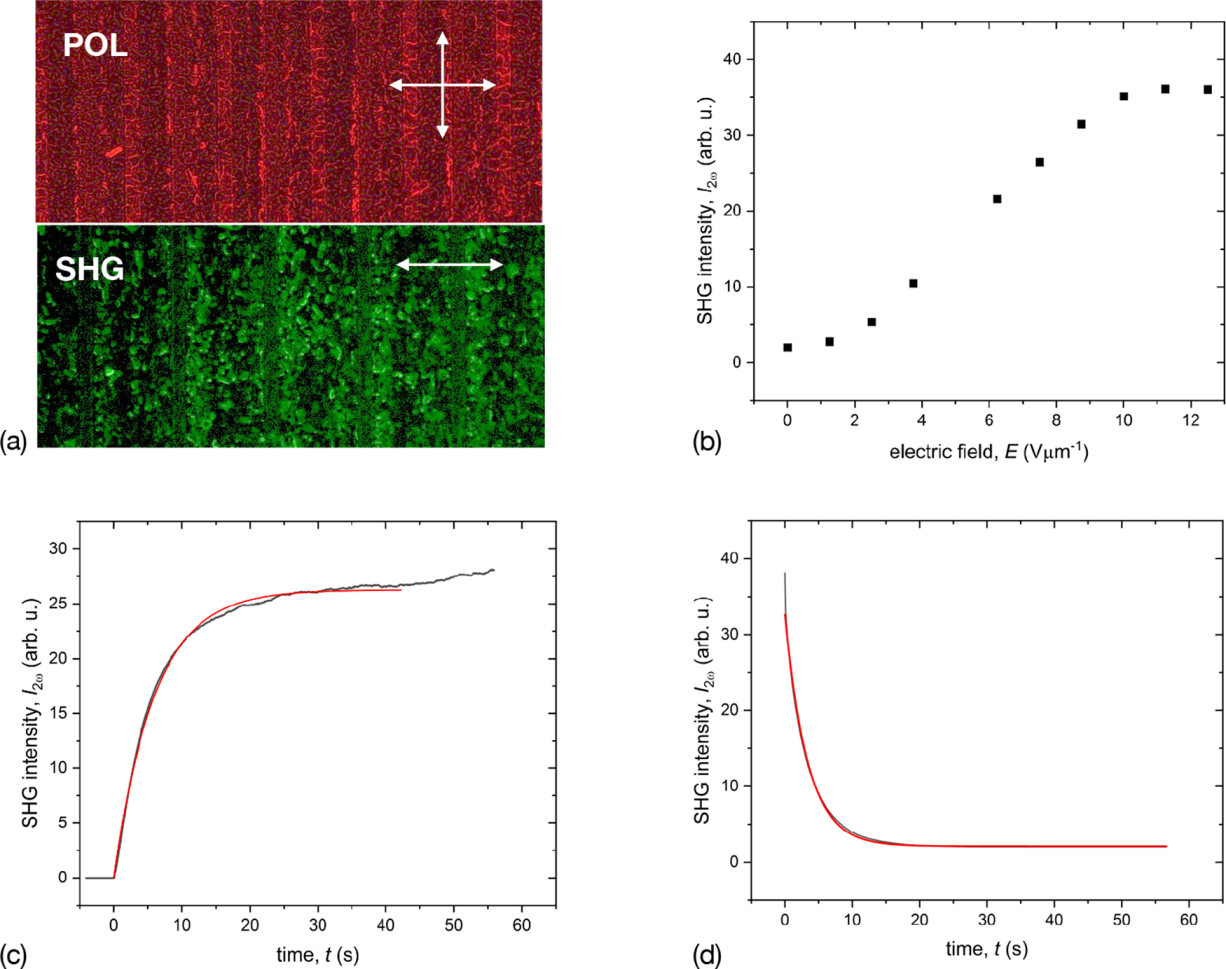
(a) Top: Polarizing microscopy texture of compound **1a** in a 6 μm thick IPS cell (*T* = 25 °C), Bottom: corresponding SHG microscopy texture recorded in the field-free state (the image width is 330 μm). The arrows indicate the orientation of the polarizer and analyzer. (b) Field dependence of the SHG signal close to the transition to the isotropic phase, (c) and (d) kinetics of the SHG induction, and relaxation respectively, in response to the application of 25 V μm^–1^. The red curves are single exponential fits with a rise time of 6.0 s, and the relaxation time of 3.4 s. The measurements were performed at *T* = 75 °C for (b–d).

The angular dependences of the SHG signal polarization for parallel and crossed orientations of the polarizers are shown in Figure 4a,b. The maximum intensity of *I*^2ω^(θ) for the extraordinary ray occurs perpendicular to the electrodes (along the polar axis), suggesting that the nonlinear optical coefficient *d*_33_ ≫ *d*_31_ (assuming *C*_*∞v*_ symmetry of the phase). The polarization of the *I*^2ω^(0) is parallel to the field (Figure 4b). Figure 5c,d highlights the kinetics of the SHG signal during switching on and off of an electric field, measured in the vicinity of the Col_h_-iso transition for compound **1a**. A combination of SHG and polarizing optical microscopies on sample cells with in-plane electrodes (IPS) allowed us to observe the textural transformation and SHG simultaneously. When an electric field was applied, birefringence was induced between the electrodes, accompanied by the SHG. The SHG’s field dependence in the vicinity of the Col_h_-isotropic transition showed a sigmoidal form reaching saturation at about 10 Vμm^–1^ at *T* = 75 °C (Figure 5b). By applying a step voltage of 25 Vμm^–1^, we could induce the SHG signal. Its kinetics could be approximated by an exponential function (Figure 5c). The characteristic rise time is 6.0 s, and the relaxation to the initial state occurred with a shorter time constant of 3.4 s (Figure 5d). As the temperature decreased, the relaxation slowed down and a state with residual polarization developed, which persisted down to room temperature (Figure 5a). The residual SHG signal displayed only a weak temperature dependence and disappeared close to the transition to the isotropic phase.

**Figure 6:**
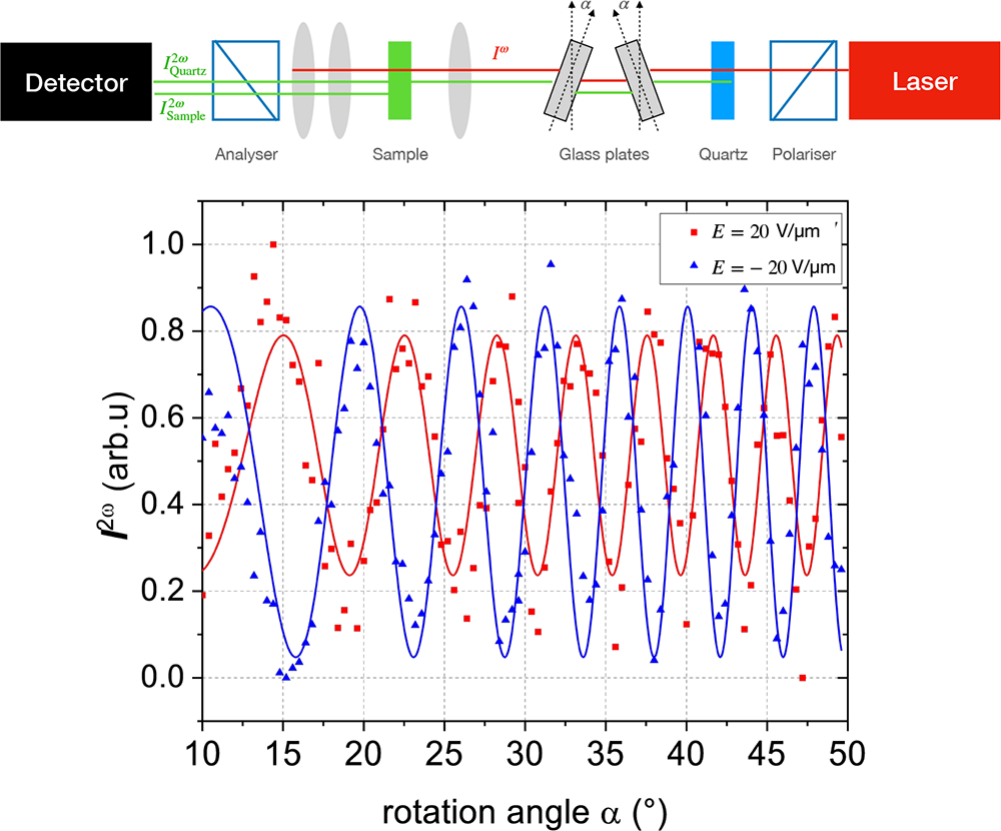
SHG interferogram recorded for compound **1b** in a 2.5 μm thick cell for aligning fields of 20 and −20 V μm^–1^.

No SHG activity was observed in the samples aligned by applying an AC electric field at a frequency above 1 Hz. When aligned with a DC field, the samples exhibited a stable SH generation, retained in a wide temperature range after field removal. The polar character of the induced state was established with the help of SHG interferometry. An interferogram recorded for compound **1b** is shown in Figure 6. Changing the polarity of the aligning field resulted in a 180° phase shift of the interferogram, confirming the excpected inversion of the polar axis.

### 3.3 Photovoltaic Behavior

**Figure 7:**
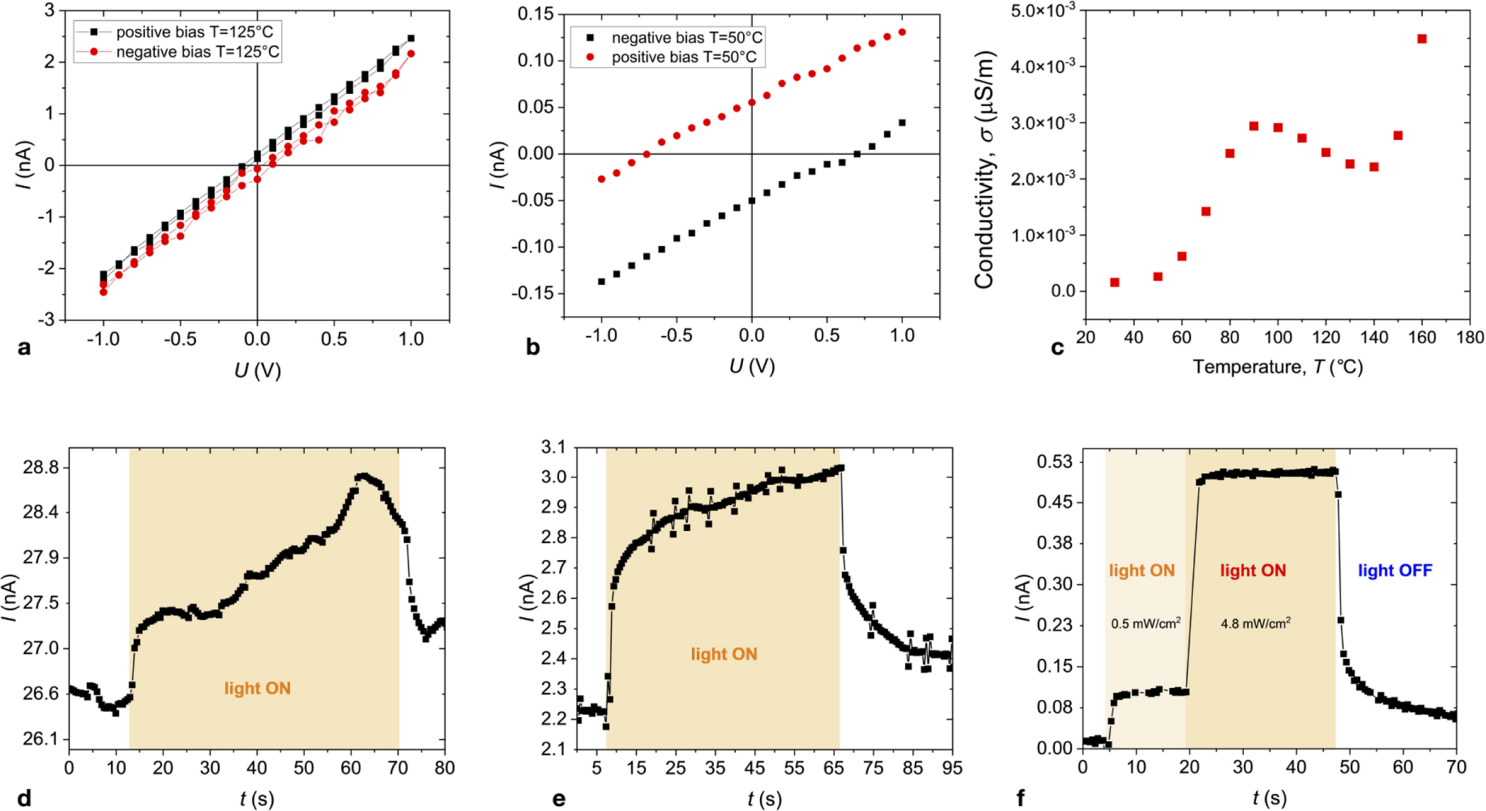
(a) *I*–*V* characteristic of compound **1b** in the isotropic phase at *T* = 125 °C. (b) Splitting of the *I*–*V* characteristics for the samples obtained by cooling under an electric bias of ±12 V μm^–1^. (c) Temperature dependence of the electric conductivity. (d–f) Photocurrent recorded under a tungsten light source (5 mW cm^–2^) at selected temperatures *T* = 120 °C (d), *T* = 100 °C (e), and *T* = 50 °C (f), respectively.

The electronic properties of the mesophase are directly influenced by its polar structure, which is reflected in the current–voltage characteristics of LC devices. When the initial state was prepared without an external electric field, the *I*–*V* was linear and showed no significant hysteresis or zero-voltage current (Figure 7a). In pure compounds, the overall illumination did not affect the *I*–*V* characteristics. However, when the initial state was prepared by cooling from the isotropic phase in an electric field, the *I*–*V* line shifted depending on the polarity of the applied voltage, as illustrated in Figure 7b. At high temperatures, ionic conductance makes a dominant contribution to the *I*–*V* characteristics. As the temperature decreases, the ionic mobility decreases, resulting in a significant reduction of the ionic conductivity (Figure 7c).

A small current of 50 pA was observed at a zero voltage in compound **1b**. Upon poling the sample in the isotropic phase and cooling it down in the electric field, the sign of the current reversed. This implies that the polar molecular columnar assemblies were aligned in the electric field’s presence, causing the current to flow in a biased direction.

Due to the broad absorption band of the compound between 250 and 660 nm,^20^ the exposure to light, even in the visible spectral range (tungsten light source), results in photocurrent generation (Figure 7d–f). At high temperatures, close to the isotropic-Col_h_ transition, ionic conductivity dominates, and it is difficult to discern a small photocurrent (Figure 7d). At lower temperatures, the dark current decreases and the photocurrent becomes more pronounced (Figure 7f). Figure 7f displays a typical response to illumination from a tungsten source at two different intensities. At *T* = 50 °C, illumination by light with 4.8 mW cm^–2^ results in the ratio of the photocurrent (IL) to dark current (ID): IL/ID = 50 with a photocurrent reaching 500 pA cm^–2^. The rise time becomes faster at lower temperatures. The low conductivity and weak photocurrent can be attributed to the deficiency of charge carriers.

**Figure 8:**
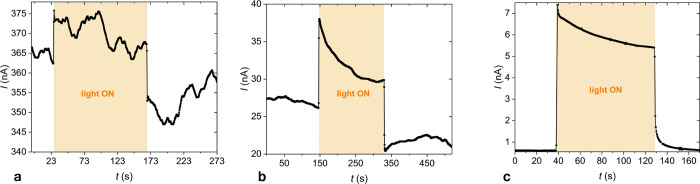
Photocurrent in C61-doped Compound **1b** recorded under a tungsten light source (5 mW cm^–2^) at selected temperatures *T* = 120 °C (a), *T* = 100 °C (b), and *T* = 50 °C (c), respectively.

**Figure 9:**
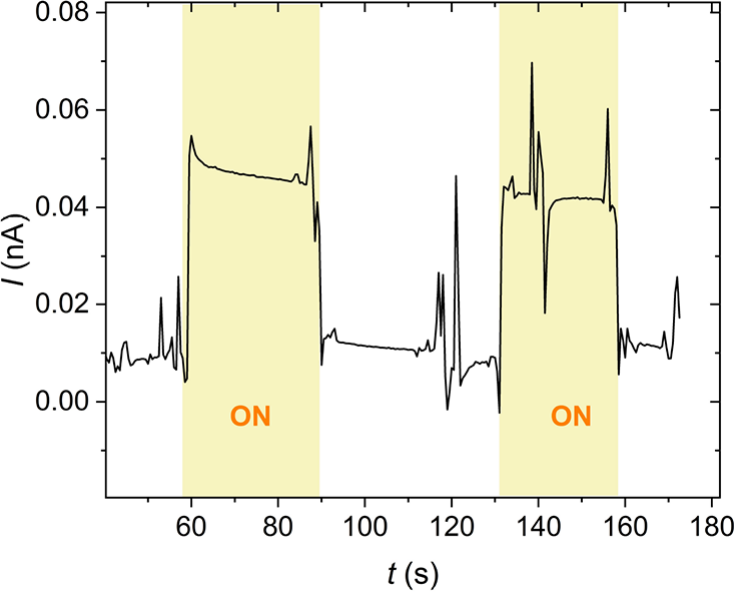
Photocurrent observed at *U* = 0 upon illumination with light (4.9 mW/cm^2^, compound **1b**).

This problem was solved by doping the liquid crystal with fullerenes ([6,6]-phenyl C61 butyric acid methyl ester (C61)). C61 is a remarkable electron acceptor successfully used in various organic electronic devices.^12,28^ Indeed, by adding C61 at a concentration of just 10 mol %, the photocurrent increased by over 1 order of magnitude, which can be seen by comparing Figures 7 and 8. The photocurrent exhibited a weak temperature dependence. At high temperatures, the photocurrent was superimposed on the ionic contribution, which strongly fluctuated with time. On cooling, the ionic conductivity decreased due to an increase of the viscosity. However, the photocurrent remained nearly constant at about 10 nA cm^–2^. A substantial decrease in photocurrent was observed below 60 °C and could be attributed to a reduction of the columnar order and the development of defects in the structure. There is a clear indication of the photocurrent generated by the internal electric field of the polarized state. Photovoltaic effect was observed at *U* = 0 in compound **1b** the field-aligned state Figure 9. Inverting the aligning field leads to a reversal of the photocurrent.

**Figure 10:**
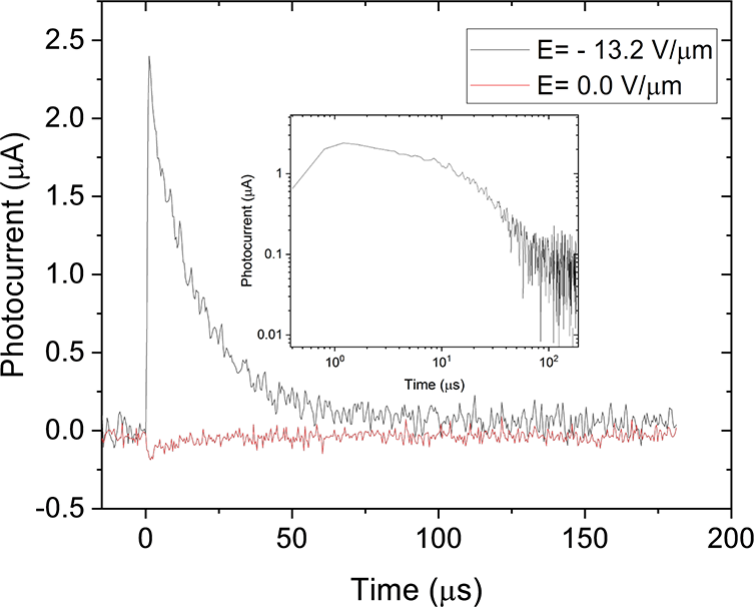
ToF transits in C61-doped compound **1b**. The black curve is recorded in an electric field of *E* = 13.2 V μm^–1^. The red curve shows the photocurrent observed at *E* = 0.

The broad absorption band of the liquid crystal allows the charge carriers to be excited in the visible range. ToF measurement performed using 532 nm excitation showed a hole mobility of 3.0 × 10^–4^ cm^2^V^–1^s^–1^. The ToF signal had a dispersive form. Interestingly, a small inverted residual ToF signal remained when the external field was removed (Figure 10). This signal can be attributed to the scattering field produced by the residual polarization. The residual electric field persisted after the external electric field was turned off. However, the signal weakened over time, as ionic impurities diffused away from the electrode region and reduced the residual electric field. Since the signal was too weak, it was difficult to determine the charge mobility.

**Figure 11:**
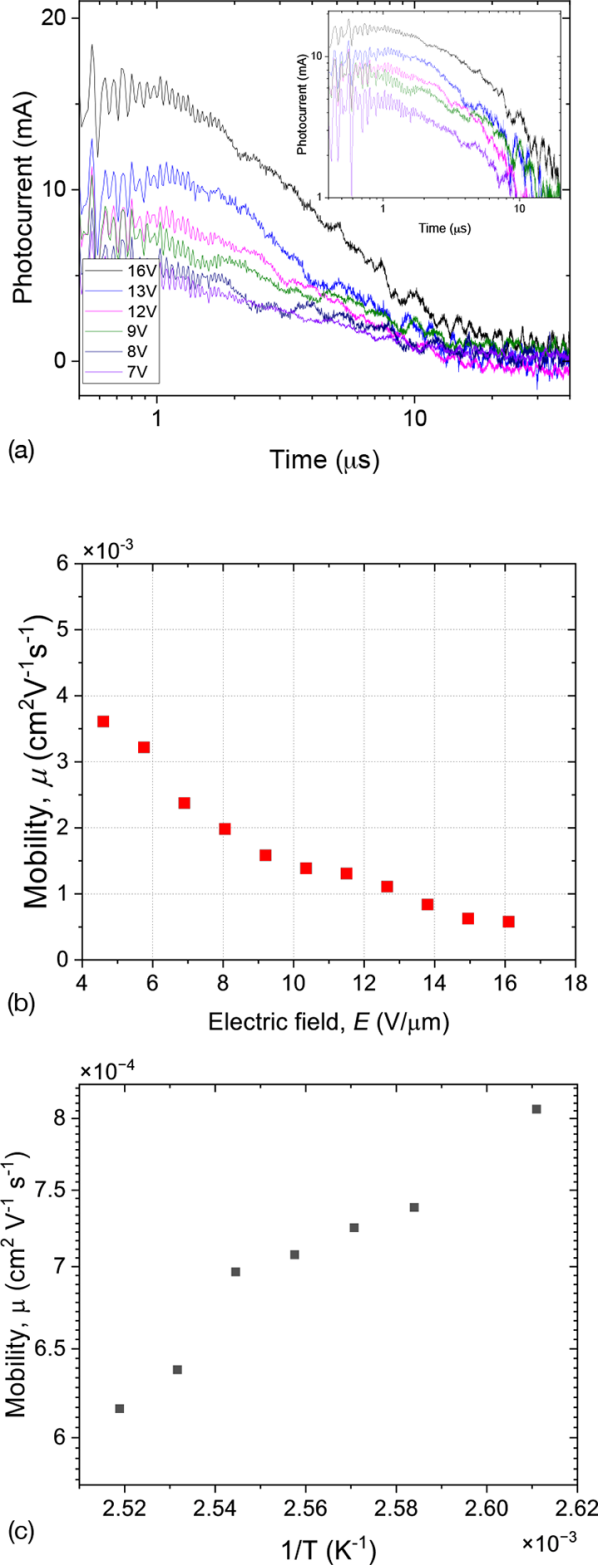
Studies of charge mobility in compound **1b**: (a) Photocurrent recorded at λ = 350 nm for different applied voltages. The inset displays the photocurrent in a double logarithmic scale. The field (b) and temperature (c) dependence of the charge mobility.

In nondoped compounds, the ToF measurements were performed at λ = 350 nm and the results are shown in Figure 11. The ToF signal has a dispersive character with typical ToF times τ_TOF_ in the range of microseconds for an applied voltage of 50–80 V (Figure 11a). However, the voltage dependence τ_TOF_ (*U*) strongly deviates from the ∝ *U*^–1^ law, giving an anomalous field-dependent mobility as shown in Figure 11b. For crystals, the field dependence of the charge mobility on the electric field *E* can be described by the Poole–Frenkel model where .

The effects of the electric field on the charge mobility in organic semiconductors have been studied in various systems. Theoretical models have been proposed, considering the field’s effects on the effective trap depth, energetic, and positional disorder.^29−32^

In general, the charge mobility increases as the electric field increases due to the decrease of the effective depth of the charge traps. At higher fields, the tunneling of the charge carriers from the traps occurs more easily as described by the Poole–Frenkel model,^33^ where the charge mobility μ increases with increasing electric field *E* as
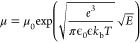
1where μ_0_ is the field-free mobility, *e* is the transferred charge, ϵ is the dielectric permittivity of the material, *k*_b_ is the Boltzmann constant, and *T* is the temperature. Energetic (diagonal) disorder attributed to the fluctuations of the lattice polarization energies is decreasing with increasing field.

However, we observed the opposite effect in our experiments, which cannot be explained by the Poole–Frenkel model. In various polymeric systems, a decrease in mobility has also been observed when they reach the low-field saturation regime. The reduction of the charge mobility can be explained as a result of the positional (off-diagonal) disorder resulting in the fluctuation of the overlapping integral and reducing the hopping rates of the charge carriers.^30,34^ Fluctuating the overlapping integral results in the charge diffusing over disordered paths, which may be partially aligned opposite to the applied electric field. In this case, the external field reduces the hopping rates, leading to the suppression of charge diffusion. The simulations demonstrated the validity of this theoretical approach, and it was shown experimentally in 1,1-bis(di-4-tolylaminophenyl)cyclohexane.^35^

However, those models do not account for the structural changes that can be driven by external electric fields. The local electric field also affects the mobility of the charge carriers. A decrease in the dipolar disorder in the ferroelectric structure will result in a reduction in the local field.

## 4 Conclusions

We studied the bulk photovoltaic properties and polar structure of umbrella-shaped subphthalocyanine-based liquid crystals. The conical shape of these mesogens promotes their self-assembly into columns distinguished by polar order. As a result, these materials exhibit a polar structure and pronounced optical nonlinearity. Although no polarization switching was observed well below the iso-Col_h_ transition, the polar state could be produced by slowly cooling the sample in an electric field from the isotropic phase. SHG interferometry studies have confirmed that polarity inversion occurs when the aligning field is inverted.

The polar structure of the mesophase determines the bulk photovoltaic properties. However, the deficiency of charge carriers results in a relatively weak photovoltaic response. Doping the material with fullerene derivatives allowed us to enhance the photocurrents significantly and have a more efficient photoelectric conversion at longer wavelengths (532 nm). Due to the residual polarization, the photocurrent could be produced without an external field applied. Still, the charge mobility is relatively low and exhibits an anomalous field dependence, decreasing considerably with increasing field strength. The nature of this effect is not entirely understood and can be attributed either to structural disorder or to the effect of the local fields.

## References

[ref1] Matsui H.; Takeda Y.; Tokito S. Flexible and printed organic transistors: From materials to integrated circuits. Org. Electron. 2019, 75, 105432 10.1016/j.orgel.2019.105432.

[ref2] Malliaras G. G.. Organic bioelectronics: A new era for organic electronics. Biochimica et Biophysica Acta (BBA) - General Subjects 2013, 1830, 4286–4287. 10.1016/j.bbagen.2012.10.007. 23079584

[ref3] Lupo D.; Clemens W.; Breitung S.; Hecker K. Applications of Organic and Printed Electronics. Integrated Circuits and Systems 2013, 1–26. 10.1007/978-1-4614-3160-2_1.

[ref4] Kato T.; Yoshio M.; Ichikawa T.; Soberats B.; Ohno H.; Funahashi M. Transport of ions and electrons in nanostructured liquid crystals. Nat. Rev. Mater. 2017, 2, 17001 10.1038/natrevmats.2017.1.

[ref5] Mayoral M. J.; Torres T.; González-Rodríguez D. Polar columnar assemblies of subphthalocyanines. J. Porphyrins Phthalocyanines 2020, 24, 33–42. 10.1142/S1088424619300167.

[ref6] Herbst S.; Soberats B.; Leowanawat P.; Stolte M.; Lehmann M.; Würthner F. Self-assembly of multi-stranded perylene dye J-aggregates in columnar liquid-crystalline phases. Nat. Commun. 2018, 9, 2646 10.1038/s41467-018-05018-6. 29980743 PMC6035248

[ref7] Iino H.; Usui T.; Hanna J.-I. Liquid crystals for organic thin-film transistors. Nat. Commun. 2015, 6, 6828 10.1038/ncomms7828. 25857435 PMC4403349

[ref8] Funahashi M. Nanostructured liquid-crystalline semiconductors - a new approach to soft matter electronics. Journal of Materials Chemistry C 2014, 2, 7451–7459. 10.1039/C4TC00906A.

[ref9] Kato T. From Nanostructured Liquid Crystals to Polymer-Based Electrolytes. Angewandte Chemie - International Edition 2010, 49, 7847–7848. 10.1002/anie.201000707. 20677293

[ref10] Kato T. Self-assembly of phase-segregated liquid crystal structures. Science 2002, 295, 2414–2418. 10.1126/science.1070967-a. 11923528

[ref11] Funahashi M. High open-circuit voltage under the bulk photovoltaic effect for the chiral smectic crystal phase of a double chiral ferroelectric liquid crystal doped with a fullerene derivative. Materials Chemistry Frontiers 2021, 5, 8265–8274. 10.1039/D1QM01143J.

[ref12] Funahashi M. Bulk photovoltaic effect in ferroelectric liquid crystals comprising of quinquethiophene and lactic ester units. Org. Electron. 2023, 122, 106911 10.1016/j.orgel.2023.106911.

[ref13] Sergeyev S.; Pisula W.; Geerts Y. H. Discotic liquid crystals: a new generation of organic semiconductors. Chem. Soc. Rev. 2007, 36, 1902–1929. 10.1039/b417320c. 17982517

[ref14] Dechant M.; Lehmann M.; Uzurano G.; Fujii A.; Ozaki M. AA. Journal of Materials Chemistry C 2021, 9, 5689–5698. 10.1039/D1TC00710F.

[ref15] Claessens C. G.; González-Rodríguez D.; Rodríguez-Morgade M. S.; Medina A.; Torres T. Subphthalocyanines, Subporphyrazines, and Subporphyrins: Singular Nonplanar Aromatic Systems. Chem. Rev. 2014, 114, 2192–2277. 10.1021/cr400088w. 24568182

[ref16] Lavarda G.; Labella J.; Martínez-Díaz M. V.; Rodríguez-Morgade M. S.; Osuka A.; Torres T. Recent advances in subphthalocyanines and related subporphyrinoids. Chem. Soc. Rev. 2022, 51, 9482–9619. 10.1039/D2CS00280A. 36354343

[ref17] Kang S. H.; Kim K.; Kang Y.-S.; Zin W.-C.; Olbrechts G.; Wostyn K.; Clays K.; Persoons A. Novel columnar mesogen with octupolar optical nonlinearities: synthesis, mesogenic behavior and multiphoton-fluorescence-free hyperpolarizabilities of subphthalocyanines with long aliphatic chains. Chem. Commun. 1999, 17, 1661–1662. 10.1039/a904254g.

[ref18] Zhang C.; Nakano K.; Nakamura M.; Araoka F.; Tajima K.; Miyajima D. Noncentrosymmetric Columnar Liquid Crystals with the Bulk Photovoltaic Effect for Organic Photodetectors. J. Am. Chem. Soc. 2020, 142, 3326–3330. 10.1021/jacs.9b12710. 32024364

[ref19] Guilleme J.; Aragó J.; Ortí E.; Cavero E.; Sierra T.; Ortega J.; Folcia C. L.; Etxebarria J.; González-Rodríguez D.; Torres T. A columnar liquid crystal with permanent polar order. Journal of Materials Chemistry C 2015, 3, 985–989. 10.1039/C4TC02662D.

[ref20] Lehmann M.; Baumann M.; Lambov M.; Eremin A. Parallel Polar Dimers in the Columnar Self-Assembly of Umbrella-Shaped Subphthalocyanine Mesogens. Adv. Funct. Mater. 2021, 31, 2104217 10.1002/adfm.202104217.

[ref21] Gorbunov A. V.; Iglesias M. G.; Guilleme J.; Cornelissen T. D.; Roelofs W. S. C.; Torres T.; González-Rodríguez D.; Meijer E. W.; Kemerink M. Ferroelectric self-assembled molecular materials showing both rectifying and switchable conductivity. Sci. Adv. 2017, 3, e1701017 10.1126/sciadv.1701017. 28975150 PMC5621973

[ref22] Rodríguez-Morgade M. S.; Claessens C. G.; Medina A.; González-Rodríguez D.; Gutiérrez-Puebla E.; Monge A.; Alkorta I.; Elguero J.; Torres T. Synthesis, Characterization, Molecular Structure and Theoretical Studies of Axially Fluoro-Substituted Subazaporphyrins. Chem. −A Eur. J. 2008, 14, 1342–1350. 10.1002/chem.200701542. 18165956

[ref23] Lehmann M.; Dechant M.; Lambov M.; Ghosh T. Free Space in Liquid Crystals-Molecular Design, Generation, and Usage. Acc. Chem. Res. 2019, 52, 1653–1664. 10.1021/acs.accounts.9b00078. 31135131

[ref24] Bloembergen N. Nonlinear Optics; World Scientific: Harvard, 1996.

[ref25] Wolff J. J.; et al. Dipolar NLO-phores with large off-diagonal components of the second-order polarizability tensor. Adv. Mater. 1997, 9, 138–143. 10.1002/adma.19970090209.

[ref26] Choi S.; Kinoshita Y.; Park B.; Takezoe H.; Niori T.; Watanabe J. Second-harmonic generation in achiral bent-shaped liquid crystals. Japanese Journal Of Applied Physics Part 1-Regular Papers Short Notes & Review Papers 1998, 37, 3408–3411. 10.1143/JJAP.37.3408.

[ref27] Araoka F.; Thisayukta J.; Ishikawa K.; Watanabe J.; Takezoe H. Polar structure in a ferroelectric bent-core mesogen as studied by second-harmonic generation. Phys. Rev. E 2002, 66, 021705 10.1103/PhysRevE.66.021705. 12241194

[ref28] Guldi D. M.; Illescas B. M.; Atienza C. M.; Wielopolski M.; Martín N. Fullerene for organic electronics. Chem. Soc. Rev. 2009, 38, 1587–1597. 10.1039/b900402p. 19587954

[ref29] Fishchuk I. I.; Kadashchuk A.; Ullah M.; Sitter H.; Pivrikas A.; Genoe J.; Bässler H. Electric field dependence of charge carrier hopping transport within the random energy landscape in an organic field effect transistor. Phys. Rev. B 2012, 86, 045207 10.1103/PhysRevB.86.045207.

[ref30] Bässler H. Charge Transport in Disordered Organic Photoconductors a Monte Carlo Simulation Study. Physica Status Solidi (b) 1993, 175, 15–56. 10.1002/pssb.2221750102.

[ref31] Asadullayev N.; Brandt N.; Chudinov S.; Kozlov S.; Ciric I. Hopping conductivity in amorphous silicon nitride in high electric and magnetic fields. Solid State Commun. 1987, 61, 511–514. 10.1016/0038-1098(87)90157-8.

[ref32] Levin E.; Shklovskii B. Negative differential conductivity of low density electron gas in random potential. Solid State Commun. 1988, 67, 233–237. 10.1016/0038-1098(88)90607-2.

[ref33] Frenkel J. On Pre-Breakdown Phenomena in Insulators and Electronic Semi-Conductors. Phys. Rev. 1938, 54, 647–648. 10.1103/PhysRev.54.647.

[ref34] Toman P.; Menšík M.; Pfleger J. Electric field dependence of charge mobility in linear conjugated polymers. Chemical Papers 2018, 72, 1719–1728. 10.1007/s11696-018-0448-0.

[ref35] Borsenberger P. M.; Pautmeier L.; Bässler H. Charge transport in disordered molecular solids. J. Chem. Phys. 1991, 94, 5447–5454. 10.1063/1.460506. 10008726

